# A study on usefulness of a set of known risk factors in predicting maternal syphilis infections in three districts of Western Province, Zambia

**DOI:** 10.11604/pamj.2016.24.75.8425

**Published:** 2016-05-24

**Authors:** Jacob Sakala, Nellisiwe Chizuni, Selestine Nzala

**Affiliations:** 1 Department of Public Health, University of Zambia, School of Medicine, Lusaka, Zambia; 2 Kaoma District Medical Office, Ministry of Health, Kaoma, Zambia

**Keywords:** Zambia, maternal syphilis, RPR, assessment criteria

## Abstract

**Introduction:**

Despite roll-out of cost-effective point-of-care tests, less than half antenatal attendees in rural western Zambia are screened for syphilis. This study formulated a clinical, risk-based assessment criteria and evaluated its usefulness as a non-biomedical alternative for identifying high-risk prenatal cases.

**Methods:**

We conducted a cross-sectional survey of antenatal clinic attendees in Kaoma, Luampa and Nkeyema districts to collect data on exposure to nine pre-selected syphilis risk factors. These factors were classified into major and minor factors based on their observed pre-study association strengths to maternal syphilis. Clinical disease was defined as exposure to either two major factors, one major with two minor factors or three minor factors. Sensitivity, specificity and predictive values of the clinical protocol were then calculated in comparison to rapid plasmin reagin results.

**Results:**

The observed syphilis prevalence was 9.3% (95% CI: 7.4 - 11.6%) and the overall sensitivity of the study criteria was 62.3% with positive predictive value of 72.9%. Sensitivities of individual case-defining categories were even lower; from 17.4% to 33.3%. Results confirmed that abortion history, still birth, multiple sexual partners, previous maternal syphilis infection, partner history of sexually transmitted infection and maternal co-morbid conditions of HIV and genital ulcer disease were significantly associated to maternal syphilis in study population as well.

**Conclusion:**

The criteria was not as effective as biomedical tests in identifying maternal syphilis. However, it could be a useful adjunct/alternative in antenatal clinics when biomedical tests are either inadequate or unavailable.

## Introduction

An estimated 11 million people are infected with syphilis globally every year; of these 1.5 million are pregnant women [1]. Maternal syphilis can lead to adverse pregnancy outcomes (APO) in 53.4 - 81.8% cases [2] such as spontaneous abortions, intra-uterine growth retardation, still births, premature deliveries, low-birth weight, perinatal deaths and congenital disease among new-born babies [3, 4]. To contribute towards control of maternal syphilis and its APOs, the World Health Organization (WHO) recommends antenatal screening of all pregnant women [5]. Many countries in sub-Saharan Africa (SSA) including Zambia, which have some of the highest levels of infection, introduced the WHO syphilis-screening recommendations into national antenatal care (ANC) guidelines [6, 7]. However, most of these SSA countries have challenges ensuring 90% antenatal access to syphilis screening services. In 2010, of the 27 countries in SSA that submitted a report, only Namibia achieved this target. The median coverage for syphilis screening in the reporting countries was 59% [8]. Zambia, on average, screens 44% pregnant women every year and the rural areas of the western province are among the worst affected. As a result of this, an estimated 241 prenatal syphilis infections are missed annually in three districts (Kaoma, Luampa and Nkeyema) of this province alone [9, 10]. To ensure universal access to antenatal syphilis testing, a number of studies have recommended the use of point-of-care (POC) biomedical tests [1, 11, 12]. To this effect, in 2012, Zambia introduced more cost effective and logistically simpler rapid syphilis tests (RST) [3, 6] to the existing antenatal screening guidelines that originally provided only for use of rapid plasmin reagin tests (RPR) [13]. Despite this, there has been minimal improvement in screening levels indicating existence of possible performance gaps related to health systems, political support and resources allocation for the programme [14, 15]. Understandably, to address existing performance gaps, the main focus for research in this area has been developing more cost-effective, biomedical means of screening for syphilis as infections can be asymptomatic. We argue that a need exists to find alternative methods for use in identifying women at high risk of infection when biomedical test are unavailable. Currently, ANC providers receive no guidance from the WHO and national guidelines on alternative measures to control maternal syphilis when biomedical tests are unavailable for any reason. A few studies, whilst in the process of conducting cost-effectiveness studies of biomedical testing, explored alternative control-strategies such as empirical treatment of all pregnant women [16–18] and presumptive syphilis diagnosis based on presence of genital ulcer disease (GUD) [11]. Results have shown that these alternative methods could be relatively cheaper than conventional methods, however, concerns of unnecessary over-treatment of antenatal attendees have discouraged their recommendation for general use [18]. Arising from this, we sought to explore the possibility of using a clinical protocol, based known risk factors of maternal syphilis, as an alternative syphilis identification method. The rationale was based on availability of documented evidence of risk factors associated with maternal syphilis which could be categorized in a clinical protocol. These risk factors included; a maternal history of previous infection with syphilis, history of abortion [19], history of multiple sexual partners [20], early maternal age at sexual debut [21], obstetric history of still birth delivery [22, 23], HIV co-infection [24], presence of genital ulcer disease [11, 25] and a history of sexually transmitted infection in the partner [26]. We then evaluated the sensitivity, specificity and predictive values of the designed protocol. We further, sought to review how these findings would influence performance gaps in antenatal syphilis screening guideline implementation in three districts of western province, Zambia.

## Methods

### Design and sampling procedures

We conducted a cross-sectional study in Kaoma, Luampa and Nkeyema districts, western province, Zambia from April to June 2015. This is the most populated region of the province with 122,092 inhabitants, majority of whom are women of child bearing age with annual expected pregnancies of 10,531 and average first antenatal attendances of 9,989. The region has 34 public health facilities offering ANC services. The primary sample was selected using one-stage cluster design which guided the initial selection of eight (8) health facility clusters using systematic random technique from a list public health facilities in the study region. Respondents were then recruited by selecting all consenting pregnant women attending antenatal clinic in the sampled clusters during the study period. The secondary sample included 10 health facility staff from these sites as well as 3 district health programme managers selected purposefully. We collected data on past exposure to risk factors of maternal syphilis from primary respondents. The RPR blood test performed on consenting respondents was gold standard syphilis test. We also performed a record review of; antenatal records, antenatal screening policy guidelines, district health systems in relation staff capacities and logistics management and latter included interviews of district health staff.

### Data collection

A structured interviewer-administered questionnaire was used for data collection. The tool was translated into Lozi, the main local language in the study region, and pre-tested for consistency. The regular ANC providers were recruited as research assistants and the questionnaire was administered in normal antenatal clinic setting to gain respondents’ trust especially that some questions could be considered sensitive.

### Laboratory methods

Blood screening for syphilis was done after administration of the questionnaire to minimise bias. The study provided test kits to facilities when unavailable. The test was conducted under the usual antenatal conditions using the IMMUTREP RPR, a non-treponemal flocculation syphilis test. Approximately 50µl venous whole blood sample from each consenting respondent was mixed with one free-falling drop of test antigen on a test card. The mixing was aided by rotating the test cards for 8 minutes after which the results were read.

### Proposed clinical assessment criteria

Risk factors were categorized into major factors which included: a maternal history of previous infection with syphilis, presence of genital ulcer disease, history of multiple sexual partners, HIV co-infection and minor factors; history of abortion, early maternal age at sexual debut, obstetric history of still birth delivery and neonatal death, as well as a history of sexually transmitted infection in the partner. This classification was based on observed strength of association or frequency of linkage of these risk factors to maternal syphilis infections with cut-off set at odds ratio of 5 and frequency of 10%. Clinical disease was then defined as either the presence of two (2) major risk factors or one (1) major and two (2) minor risk factors or three (3) minor risk factors.

### Data analysis

The data from the questionnaires was coded, checked and cleaned before entry into a Microsoft excel sheet and imported into Stata version 13 for analysis. Data on risk factor identification and laboratory results were assigned numbers either 1 or 2 depending on presence or absence of a risk factor or disease. These were then entered in Microsoft excel and imported into Stata version 13. Proportions were used to estimate prevalence of maternal syphilis. Since data variables of risk factors was dichotomous, univariate and multivariate analysis for binary outcomes was done to find the relationship to maternal syphilis with odds ratio and chi-square as measures of association. Multiple logistic regression was done to test for significance set at 95% confidence level with p value < 0.05. Using RPR as confirmatory test, the sensitivity, specificity and predictive values of the proposed criteria were calculated to measure its usefulness in identifying maternal infections. The accuracy of the assessment criteria was ascertained by calculating the area under the receiver operating curve (ROC) which compared ability of a test to differentiate between those with disease and those without. A ROC of greater than 0.80 was deemed to have good accuracy, while 0.70 to 0.80 was fair and less than 0.70 was deemed to be poor. We used content analysis method to summarize qualitative data from desk review and health personnel unstructured interviews. This was then reported by use of narratives which in some cases included direct key quotations.

### Ethical Considerations

Ethics approval was obtained from Excellence in Research Ethics and Science (ERES) Converge; (2014-Aug-016). Participation was consensual and data was handled confidentially.

## Results

### Population characteristics

A total 740 respondents who gave complete responses were recruited in the study. The majority; 360 (48.6%) were aged between 20 and 30 years with a mean age of 26 ± 0.5 years. The results also showed that a greater proportion of the respondents; 445 (60.1%) were married, had more than one pregnancy; 572 (77.3%) and were unlikely to have gone beyond primary level of school education; 497 (67.2%).

### Syphilis sero-positivity in study population

The syphilis sero-positivity using RPR tests was observed to be 9.3% (95% CI: 7.4 -11.6%) among study participants. Of the women testing positive, 61 (87.1%) were multigravidas as opposed to 9 (12.9%) who were in their first pregnancy.

### Correlates between risk-factors and maternal syphilis infections among respondents

A univariate comparison of risk factors to maternal syphilis showed that cases were more likely to have a history of abortion, a history of still birth delivery, to have previously lost a baby in the first month of birth, to have previous infection with syphilis, to have a sexual partner with a sexually transmitted infection, to have had multiple sexual partners in past (two) 2 years, to have genital ulcer disease and be HIV co-infected. However, the association between maternal syphilis infection and early sexual debut (before the age of 16 years) at this stage was found not to be statistically significant as the chi-square test for this association was found to have a P-value greater than 0.05 (Table 1). We then conducted a multivariate logistics analysis excluding early age at sexual debut. After controlling for all variables we found that co-morbid conditions of HIV and genital ulcer disease and exposure histories of still birth delivery and previous infection with syphilis were strongly associated with syphilis sero-positivity (OR > 5). Other risk factors such as history of abortion, having more than one sexual partners and sexually transmitted infection in a sexual partner were also significantly associated with gestational syphilis infections (OR:3 to 5). At this stage, we also found that a history of losing a neonate through death was not significantly associated to maternal syphilis (OR 2.3, p value> 0.05) (Table 2). Therefore only seven (7) of the nine (9) preselected factors in the end were found to be significantly associated with maternal syphilis.

**Table 1:** Univariate analysis: association between risk factors and maternal syphilis

Variable	RPR positive Number* (%)	RPR negative Number* (%)	OR (95% CI)	P value
**Abortion history**				
No	39 (66.1%)	478 (93.4%)	1.00	
Yes	20(33.9%)	34 (6.6%)	7.21 (3.80-13.69)	<0.001
Still birth history				
No	44 (77.2%)	493 (96.3%)	1.00	
Yes	13 (22.8%)	19 (3.7%)	7.67 (3.55-16.56)	<0.001
**Neonatal death history**				
No	50 (86.2%)	495 (95.6%)	1.00	
Yes	8 (13.8%)	23 (4.4%)	3.44 (1.46-8.10)	0.003
**Previous Syphilis infection**				
No	49 (71.0%)	641 (97.7%)	1.00	
Yes	20 (29.0%)	15 (2.3%)	17.44 (8.41-36.18)	<0.001
**Genital ulcer disease**				
No	52 (76.5%)	652 (97.9%)	1.00	
Yes	16 (23.5%)	14 (2.1%)	14.3 (6.62-30.97)	<0.001
**Early sexual debut <16yrs**				
No	36 (52.2%)	424 (63.2%)	1.00	
Yes	33 (47.8%)	247 (36.8%)	1.57 (0.96-2.56)	0.072
**Multiple sexual partners**				
No	48 (69.6%)	573 (86.75%)	1.00	
Yes	21 (30.4%)	96 (14.4%)	2.6 (1.50-4.56)	<0.001
**Partner STI infection**				
No	37 (64.9%)	503 (94.2%)	1.00	
Yes	20 (35.1%)	31 (5.8%)	2.6 (1.50-4.56)	<0.001
**HIV infection**				
No	50 (72.5%)	648 (96.6%)	1.00	
Yes	19 (27.5%)	23 (3.4%)	10.7 (5.47-20.07)	<0.001

OR = Odds ratio, CI = Confidence Interval

* Not all totals sum to the recruited 740 due to missing values/non applicability of exposure factor

**Table 2:** Multivariate analysis: association of risk factors with maternal syphilis

Variable	RPR positive Number* (%)	RPR negative Number* (%)	OR (95% CI)	P value
**Abortion history**				
No	39 (66.1%)	478 (93.4%)	1.00	
Yes	20 (33.9%)	34 (6.6%)	4.5 (1.82 – 11.21)	0.001
**Still birth history**				
No	44 (77.2%)	493 (96.3%)	1.00	
Yes	13 (22.8%)	19 (3.7%)	6.4 (1.92 – 21.05)	0.002
**Neonatal death history**				
No	50 (86.2%)	495 (95.6%)	1.00	
Yes	8 (13.8%)	23 (4.4%)	2.3 (0.59 – 9.28)	0.228
**Previous Syphilis infection**				
No	49 (71.0%)	641 (97.7%)	1.00	
Yes	20 (29.0%)	15 (2.3%)	6.1 (2.07 – 17.81)	0.001
**Genital ulcer disease**				
No	52 (76.5%)	652 (97.9%)	1.00	
Yes	16 (23.5%)	14 (2.1%)	6.4 (1.68 – 24.74)	0.007
**Multiple sexual partners**				
No	48 (69.6%)	573 (86.7%)	1.00	
Yes	21 (30.4%)	96 (14.4%)	4.0 (1.56 – 10.04)	0.004
**Partner STI infection**				
No	37 (64.9%)	503 (94.2%)	1.00	
Yes	20 (35.1%)	31 (5.8%)	3.3 (1.32 – 8.26)	0.011
**HIV infection**				
No	50 (72.5%)	648 (96.6%)	1.00	
Yes	19 (27.5%)	23 (3.4%)	8.4 (3.26 – 21.49)	0.001

OR = Odds ratio, CI = Confidence Interval

Non-exposure response to risk factors reference

* Not all totals sum to the recruited 740 due to missing values/non applicability of exposure factor

### Sensitivity, specificity and predictive value of proposed risk assessment criteria

The proposed assessment criteria identified 59 (8%) of the respondents with presumptive clinical disease. Of these, 43 were true positive (TP) cases of syphilis. The criteria also identified 655 women as true negatives (TN). However, 26 (37.7%) women with disease were missed and 27.1% were incorrectly classified as diseased when they were syphilis sero-negative. The overall sensitivity of the assessment criteria was 62.3% with a positive predictive value (PPV) of 72.9% and its specificity was 97.6% with a negative predictive value (NPV) of 96.2%. The area under the receiver operating curve (ROC) at 0.780 corresponded to a fair accuracy result (Table 3). The individual case definition categories showed lower sensitivities than their combined effect. Presence of two major risk factors was more sensitive at 33.3% sensitivity, followed by the category with one major and two minor factors and the least was the category with three minor factors. The areas under the ROC for the individual case categories were all lower than 0.7 showing their reduced accuracy.

**Table 3:** Sensitivity, specificity and predictive value of proposed risk assessment criteria

Screening criteria	Frequencies	Sensitivity	Specificity	PPV	NPV	ROC
All assessment categories combined	TP:43 FP:16 FN:26 TN:655	62.3%	97.6%	72.9%	96.2%	0.780
Two major risk factors	TP:23 FP:7 FN:46 TN:664	33.3%	98.9%	76.7%	93.5%	0.662
One major and two minor risk factors	TP:20 FP:6 FN:49 TN:665	29.0%	99.1%	76.9%	93.1%	0.641
Three minor risk factors	TP:12 FP:4 FN:57 TN:667	17.4%	99.4%	75.0%	92.1%	0.584

TP = True positives. FP = False positives. TN = True negatives. FN = False negatives

PPV = Positive predictive value. NPV = Negative predictive value

### Observed Gaps in the antenatal syphilis screening guidelines

ANC guidelines require first visit syphilis screening for pregnant women, however biomedical tests availability is a challenge due to; under-supply ( in all eight facilities), irregular submission of commodity consumption data for quantification of supply (two facilities) and inadequate resource allocation at district level to supplement supply; Although syndromic STI management is practiced in STI clinics, this is not practiced in antenatal clinic and antenatal attendees are left untreated in absence of biomedical tests; All ANC frontline staff were oriented on simple technique of using RST however they were not fully aware of revision in syphilis guidelines promoting their use.

## Discussion

This study confirmed that most of the pre-selected socio-demographic, behavioural and medical risk factors were significantly associated with maternal syphilis infection even in the study population validating their inclusion in the clinical assessment protocol. Respondents with syphilis were not only more likely to have co-morbid conditions like HIV and genital ulcer disease but also reported a history of multiple sexual partners, previous abortion, previous still birth delivery, previous syphilis infection and having a sexual partner with a sexually transmitted infection. The overall sensitivity of the protocol compared well to off-site field validation tests for point of care (POC) treponemal tests conducted in a syphilis clinic in Manaus, Brazil. In this study, off-site POC tests were reported to have sensitivities in the range of 45.8 to 66.7% [27]. The sensitivity of this study's assessment criteria however, with its inherent limitations was inferior to the widely recommended on-site rapid syphilis screening tests such as treponemal-based immuno-chromatographic strips (ICS). The latter have been reported to have field sensitivities ranging from 85 to 95% [28, 29]. The possible sources of bias included information bias as it is unclear whether there were any risk factors relevant to the local study population omitted from the assessment criteria. Further, evaluation of clinical disease was essentially based on self-reported exposure to risk factors. It is likely therefore that the results may be affected by the participants’ ability and willingness to recall and disclose exposure to certain risk behaviours. Generally, the clinical protocol performed reasonably well in predicting maternal syphilis infections even though the proportion of clinically presumed infections (8%) was lower than the actual sero-positive cases (9.3%). We considered the possibility that the observed protocol's performance might be due to sample-size related over-estimation of sero-prevalence which differed considerably from routine data in the study area. The 2007 Zambia Demographic Health Survey (ZDHS) estimated 4% prevalence levels among women of reproductive age group [30] and an observational study by Makasa et al showed declining national syphilis prevalence trends in concurrence to declines in HIV prevalence in Zambia [31].

However, this study's observed maternal syphilis sero-prevalence compares well to estimates from other studies in the country which generated information from antenatal clinics. In 2014 a study to evaluate rapid Dual HIV and syphilis tests, at the University Teaching Hospital in Lusaka, showed syphilis prevalence of 9.5% among women attending antenatal clinic [32]. This was similar to what was observed by Makasa et al, when they found high sero-prevalence of 10.8% in rural sites of Western province using antenatal sentinel surveillance data [31]. It is unclear although reasonable to assume that the disease-prediction performance of the clinical protocol would be affected by prevalence level of the disease. Therefore, additional and broader studies need to be performed to evaluate variability of the criteria's results at different syphilis point prevalence levels. The difficulty in predicting syphilis infections clinically with symptoms or risk factors is one of the main reasons WHO guided national policies recommend antenatal testing for all pregnant women using biomedical tests. However, despite having a policy in place, there are still challenges in ensuring that all ANC attendees access syphilis screening services. Some of these challenges arise from weaknesses in health systems such as; ineffective laboratory commodity supply and reporting systems, partial roll-out of the more cost-effective and easier to use RST and inadequate dissemination of revised screening guidelines. The overriding challenge affecting antenatal syphilis screening is the limitations in resources allocation to ensure availability of biomedical tests. The current level of commodity supply of biomedical syphilis test in the study area does not reflect political will to adhere to recommended policy of screening “all women attending antenatal clinic”. Maintaining political will during implementation in Zambia is still a challenge despite recommendations which were accepted by the Ministry of Health to introduce point-of-care RST tests in national syphilis control guidelines [3]. This political will may diminish further as data show declining trends of syphilis prevalence [31]. There seems to be limited available alternatives to this problem. Some researchers have therefore recommended development of a dual test that would incorporate the much more politically acceptable HIV antenatal test [29] or epidemiological treatment for all pregnant women. This study's proposed clinical assessment protocol may be useful in identifying high risk infections for treatment. It could therefore be used for selective syphilis screening in declining disease-prevalence situations to limit costs of biomedical testing. It could also be used as an alternative screening tool in situations when biomedical tests are unavailable and in this situation it carries the advantage over epidemiological treatment in that sexual partners of the identified cases could also access treatment.

## Conclusion

This study was able to illustrate that a clinical assessment protocol that is based on known socio-demographic, behavioral and medical risk factors of maternal syphilis can be used to identify women at high risk of infection. Despite its diagnostic limitations the protocol offers an alternative screening method, lacking in the national syphilis control guidelines, that could be used by frontline care providers. Even though Biomedical syphilis tests remain the most cost-effective means of identifying antenatal syphilis infections, there are some challenges related to health delivery systems in Zambia that have affected regular commodity availability. However, the proposed clinical protocol could offer an acceptable means of either identifying some cases in absence of biomedical tests or prioritizing those to be screened especially in resource limited settings.

### What is known about this topic

- Biomedical tests such as RPR and RST are known to be cost-effective methods of screening for syphilis and are recommended in most National Antenatal Care Guidelines for control of maternal syphilis and its adverse outcomes;
- The national antenatal guidelines reviewed offer care providers no alternatives to biomedical testing for identifying high risk cases in limited supply settings;
- There are several socio-demographic, behavioural and medical risk factors of maternal syphilis which have not being categorized into a protocol to predict infections.

### What this study adds

- The study proposed for the first time, a clinical assessment protocol based on known risk factors of maternal syphilis for use in either guiding presumptive treatment of women at high risk of infection or as adjunct to biomedical testing in situations where screening tests are either unavailable or in limited supply respectively;
- The study tested the sensitivity of the clinical protocol which was not high enough to replace biomedical tests but sufficiently useful to be recommended for inclusion in national antenatal care guidelines as alternate/adjunct to biomedical screening.
